# Optimizing Feeding Practices Improves Outcomes During Early Childhood in Former Small for Gestational Age (SGA) Infants

**DOI:** 10.3390/nu18142296

**Published:** 2026-07-13

**Authors:** Mariana-Lăcrămioara Bucur-Grosu, Andreea Luciana Avasiloaiei, Iolanda Valentina Popa, Alexandru Burlacu, Luminița Păduraru, Alexandru Cărăuleanu, Oana-Raluca Temneanu, Demetra Gabriela Socolov

**Affiliations:** 1 Doctoral School, “Grigore T. Popa” University of Medicine and Pharmacy, 700115 Iași, Romania; mbucurgrosu@yahoo.com; 2 Department of Mother and Child Health, “Grigore T. Popa” University of Medicine and Pharmacy, 700115 Iași, Romania; luminita.paduraru@umfiasi.ro (L.P.); ale.carauleanu@umfiasi.ro (A.C.); temneanu.oana@umfiasi.ro (O.-R.T.); demetra.socolov@umfiasi.ro (D.G.S.); 3 Faculty of Medicine, “Grigore T. Popa” University of Medicine and Pharmacy, 700115 Iași, Romania; iolanda-valentina.g.popa@umfiasi.ro (I.V.P.); alexandru.burlacu@umfiasi.ro (A.B.)

**Keywords:** small-for-gestational-age, early nutrition practices, infant nutrition, breastfeeding, mixed feeding, complementary feeding, obesity, overweight, allergies

## Abstract

**Background:** Infants born small for gestational age (SGA) are at increased risk of altered postnatal growth and early metabolic vulnerability. Early feeding practices may modulate these outcomes, but evidence from Eastern Europe remains limited. **Objective:** To evaluate the influence of birth weight and early nutrition on anthropometric outcomes at 2–3 years of age in former SGA infants in Romania, and to examine associations with allergies, respiratory infections, and food intolerances. **Methods:** We conducted a retrospective comparative study including two cohorts of term SGA infants born in a Romanian tertiary hospital in 2010–2011 (n = 600) and 2020–2021 (n = 508). Perinatal data were extracted from medical records; anthropometrics and feeding practices at 2–3 years were obtained via parental telephone interviews. BMI z-scores were calculated using WHO standards. Logistic regression models were used to identify predictors of overweight/obesity and selected health outcomes. **Results:** Breastfeeding increased markedly over one decade (59.1% vs. 35.5%, *p* < 0.001), as did appropriate complementary feeding (74.2% vs. 66.3%, *p* = 0.005). Obesity prevalence decreased from 29.5% to 22.4% at 2 years and from 32.7% to 20.7% at 3 years (both *p* < 0.001), while overweight prevalence increased slightly. In the historical cohort, mixed feeding (vs. formula) predicted overweight/obesity at 2 years (aOR 2.55, 95% CI 1.52–4.43, *p* < 0.001); the association was directionally similar but not significant in the contemporary cohort (aOR 1.62, 95% CI 0.75–3.47, *p* = 0.215). Breastfeeding was protective in both cohorts. Appropriate complementary feeding was associated with lower allergy prevalence in the historical cohort (14.8% vs. 24.8%, *p* = 0.004). Food intolerances increased over time (16.3% to 27.6%, *p* < 0.001), while respiratory infections showed no significant differences. **Conclusions:** In this Romanian SGA population, early feeding practices improved substantially over one decade and were associated with more favorable weight outcomes, including a notable reduction in obesity. Mixed feeding emerged as a potential risk factor for early overweight, whereas breastfeeding remained protective. Despite these improvements, former SGA infants continue to exhibit elevated risk for early-onset overweight and obesity, underscoring the need for targeted nutritional counseling and longitudinal monitoring.

## 1. Introduction

A substantial proportion of infants born small for gestational age (SGA) have experienced fetal growth restriction (FGR), a condition resulting from inadequate fetal growth potential during intrauterine life. Globally, FGR and SGA remain major public health concerns, affecting both short-term neonatal outcomes and long-term health trajectories. SGA infants have an increased risk of perinatal morbidity and mortality, impaired neurodevelopment, metabolic dysfunction, and cardiovascular disease later in life [1,2,3]. Although not all SGA infants are pathologically growth-restricted, distinguishing constitutionally small infants from those affected by FGR remains clinically challenging.

Over the last few decades, increasing evidence has supported the Developmental Origins of Health and Disease (DOHaD) concept, which proposes that adverse intrauterine conditions may induce permanent physiological and metabolic adaptations with consequences extending into adulthood [4,5]. One of the first researchers to emphasize this relationship was Barker, through the “thrifty phenotype” hypothesis. According to this theory, fetal exposure to nutritional deprivation results in adaptive metabolic reprogramming aimed at preserving the function of vital organs, particularly the brain and heart, at the expense of peripheral tissues and long-term metabolic balance [6,7]. Although beneficial for short-term survival, these adaptations may become maladaptive when postnatal nutrition is abundant, predisposing individuals to obesity, insulin resistance, hypertension, and cardiovascular disease [8].

Following birth, many former FGR infants exhibit accelerated postnatal growth, commonly referred to as “catch-up growth”. While catch-up growth is generally considered desirable because it allows infants to approach normal anthropometric trajectories, excessive or rapid catch-up growth has been increasingly associated with adverse metabolic outcomes [9,10]. Several studies have demonstrated that rapid early weight gain in former SGA infants correlates with increased adiposity, altered body composition, elevated blood pressure, and a higher risk of metabolic syndrome later in life [11,12]. Importantly, the timing and quality of postnatal nutrition appear to significantly influence these outcomes.

Early nutrition during infancy represents one of the most important modifiable environmental factors affecting long-term metabolic health. Breastfeeding has consistently been associated with favorable metabolic and immunological effects, including improved appetite regulation, modulation of the gut microbiome, and reduced risks of obesity and allergic disease [13,14]. In contrast, formula feeding and potentially inappropriate complementary feeding practices may contribute to excessive caloric intake and altered metabolic programming. Nevertheless, evidence regarding the optimal feeding approach for former SGA infants remains incomplete and, in some respects, controversial. While adequate nutritional support is necessary to promote growth and neurodevelopment, concerns persist regarding the potential metabolic consequences of overly rapid weight gain.

In addition to metabolic outcomes, increasing attention has been directed toward the relationship between early nutrition and immune-related conditions such as allergies, food intolerances, and respiratory infections. Nutritional exposures during infancy may influence immune maturation and tolerance development through mechanisms involving intestinal barrier function, microbial colonization, and inflammatory regulation [15]. However, data specifically focusing on former SGA infants remain limited, and data regarding long-term growth patterns and feeding practices among SGA infants from Eastern European populations are mostly missing in the medical literature.

Despite the growing body of literature on FGR and metabolic programming, relatively few studies have simultaneously evaluated the influence of birth weight and early feeding practices on both anthropometric and immune-related outcomes during early childhood in SGA populations. Furthermore, temporal changes in feeding practices over the last decade may have altered postnatal growth patterns and associated risks, making cohort comparisons particularly relevant.

Our main hypothesis was that improved breastfeeding and complementary feeding over one decade would lead to better outcomes in terms of weight control in former SGA infants. The primary objective of this study was to evaluate the influence of birth weight and early feeding practices on weight gain during early childhood. The secondary objective was to investigate possible associations between early nutritional patterns and the occurrence of allergies, food intolerances, and respiratory infections during early childhood.

## 2. Materials and Methods

We performed a retrospective study in which we compared two groups of children born SGA—one “historical” group of 600 children born between 2010 and 2011 and one “contemporary” group of 508 children born between 2020 and 2021. At birth, we recorded their birth weight, length, and gestational age. At 2 or 3 years old, their parents/legal guardians were contacted for a telephone interview during which they were asked about their child’s current weight, height, and data concerning nutrition during the first year of their life, as well as health issues that arose throughout the child’s life.

Nutrition was observed in terms of the type of milk used for infant feeding and complementary feeding. Infant feeding was divided into the following: breastmilk exclusively, mixed feeding (varied proportions of breastmilk and formula), and formula feeding. Complementary feeding was evaluated in terms of timing (we considered the correct timing to be 6 months according to WHO, and we accepted ±1 month as being correct) and the type of starting food (e.g., pureed vegetables, unflavored yogurt, and mashed banana were considered appropriate; nuts, sweetened drinks, whole cow’s milk, and processed meats were considered inappropriate).

Based on their declared measurements, we calculated the children’s body mass index (BMI), and we plotted each of the values against the WHO z-score charts for 2-year-olds and 3-year-olds, respectively. We defined overweight as BMI z-score > +2 SD and obesity as BMI z-score > +3 SD, as per WHO standards for children under the age of 5 [16].

The health issues we considered were allergies, respiratory infections, and food intolerances, for which medical help was sought. The data was based on the parents’ assessment and not on the review of medical charts.

Inclusion criteria: we included former SGA singleton infants (birth weight below -2SD for their respective gestational age) with gestational ages ≥36 weeks that were born in our hospital and were admitted to the Healthy Neonatal Ward.

Exclusion criteria: we excluded from our study SGA infants with a GA < 36 weeks, infants from multiple pregnancies, infants that for any reason needed admittance to the Neonatal Intensive Care Unit, or that presented any congenital malformation.

This study was approved by the Institutional Review Board of “Grigore T. Popa” University of Medicine and Pharmacy (no. 250/27 December 2022). Informed consent was obtained at the beginning of the recruitment process from the parents/guardians of all subjects involved in the study.

### Statistical Methods

All statistical analyses were performed in R version 4.5.2 using RStudio IDE version 2025.09.0 (Posit Software, Boston, MA, USA). Multiple imputation by chained equations was performed using the mice package version 3.18.0. Logistic regression models, chi-square tests, Wilcoxon rank-sum tests, and correlation analyses were performed using functions from the *stats* package version 4.5.2. Regression model outputs were processed using *broom* version 1.0.10. Data manipulation and preparation were performed using *dplyr* version 1.1.4, *tidyr* version 1.3.1, *janitor* version 2.2.1, and *tibble* version 3.3.0. Figures were generated using *ggplot2* version 4.0.0.

Continuous variables were summarized as median and interquartile range (IQR), whereas categorical variables were presented as absolute frequencies and percentages.

Missing data were handled using multiple imputation by chained equations. Imputed values were generated over 20 iterations after excluding identifier variables and variables with no variability. The imputed dataset was subsequently used for comparative and regression analyses. Variable-level missingness for all variables included in the imputation model is summarized in Supplementary Table S1.

Comparisons between the 2010–2011 and 2020–2021 cohorts for continuous variables were performed using the Wilcoxon rank-sum test, given their non-normal distribution, while categorical variables were compared using the chi-square test.

To further investigate the association between feeding practices and clinical outcomes, logistic regression analyses were performed separately for each cohort. The dependent variables were overweight/obesity at 2 years of age, allergies, respiratory infections, and food intolerances, while the independent variables included feeding type and sex. Results were expressed as odds ratios (ORs) with 95% confidence intervals (95% CIs) and corresponding *p*-values.

Associations between early-life exposures and later anthropometric outcomes were assessed using statistical methods chosen according to variable type. Pearson’s correlation coefficient was used for associations between continuous variables, specifically birth weight versus weight at 2 and 3 years, and birth length versus height at 2 and 3 years. Spearman’s rank correlation coefficient was used for associations between infant feeding type and continuous anthropometric outcomes, as feeding type was treated as an ordinal variable. For complementary feeding, coded as a binary variable (appropriate vs. inappropriate), point-biserial correlation coefficients were calculated in relation to continuous anthropometric outcomes.

All statistical tests were two-sided, and statistical significance was defined as a *p*-value < 0.05. For clarity of presentation, *p*-values below 0.001 were reported as *p* < 0.001.

## 3. Results

The contemporary cohort (group C) demonstrated significantly increased perinatal auxological parameters compared to the historical cohort (group H), with higher gestational age (median 38.8 vs. 37.8 weeks), birth weight (2.73 vs. 2.31 kg), and birth length (48.4 vs. 47.5 cm), all with *p* < 0.001 (Table 1).

**Figure 1:**
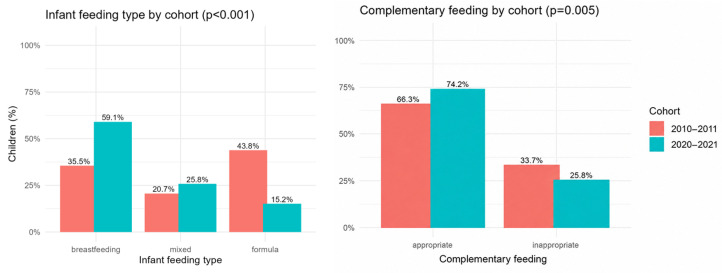
Infant feeding during the first 6 months (**left**) and start of complementary feeding (**right**) for the two groups.

Regarding feeding types until 6 months of age (Figure 1), there is a marked shift towards breastfeeding in the contemporary cohort compared to the historic one (59.1% vs. 35.5%). Also, any feeding that included breastmilk was preferred in the contemporary cohort, as it is observable in the mixed feeding group (25.8% vs. 20.7%), although to a lower extent.

Complementary feeding also improved in the contemporary cohort, as confirmed by the higher percentage of appropriate feeding (74.2% versus 66.3%, *p* = 0.005).

**Figure 2:**
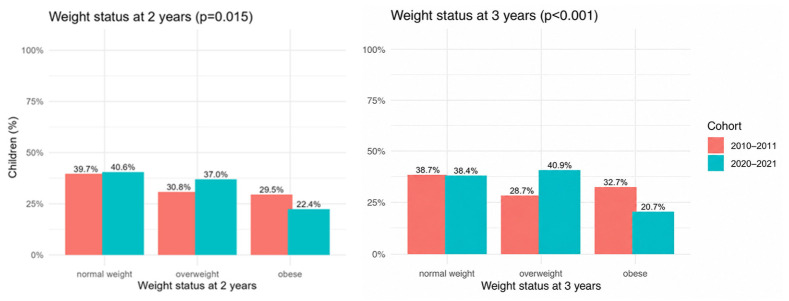
Weight status at 2 (**left**) and 3 (**right**) years of age.

In both cohorts (Figure 2), the percentage of children with a normal weight at ages 2 and 3 remained constant. There is a slight increase in overweight children in the contemporary cohort at both 2 and 3 years of age, but a substantial decrease in obesity, from 29.5% to 22.4% (−7.1 percentage points) in 2-year-olds and from 32.7% to 20.7% (−12 percentage points) in 3-year-olds.

The incidence of allergies was higher in children who had inappropriate CF in the historical cohort (24.8% vs. 14.8%, *p* = 0.004). To evaluate whether the association between CF and allergies differed by cohort, we fitted a pooled adjusted logistic regression model including a cohort × CF interaction term. Inappropriate CF was associated with higher adjusted odds of allergies in both cohorts (2010–2011: aOR 2.99, 95% CI 1.91–4.67; 2020–2021: aOR 2.83, 95% CI 1.76–4.54). The interaction term was not statistically significant (*p* = 0.868), indicating no evidence of cohort-specific effect modification (Supplementary Tables S7–S9).

An exploratory feeding type × sex interaction analysis for allergy outcomes showed no statistically significant interaction in either cohort (2010–2011: *p* = 0.111; 2020–2021: *p* = 0.695). These findings suggest that the association between feeding type and allergies did not differ by sex, although some sex-by-feeding subgroups were small and results should be interpreted cautiously.

Respiratory infections were found less often in children with appropriate CF in both cohorts, but the results did not show any statistically significant differences (2010–2011: 10.5% vs. 12.6%, *p* = 0.546; 2020–2021: 14.0% vs. 14.6%, *p* = 0.977). Regarding food intolerances, there were no differences between the children that received appropriate CF versus the ones that were inappropriately fed in either cohort (2010–2011: 16.3% vs. 12.4%, *p* = 0.246; 2020–2021: 27.6% vs. 28.2%, *p* = 0.975), although it is interesting to observe that the overall incidence of food intolerances has almost doubled in the given timeframe.

**Figure 3:**
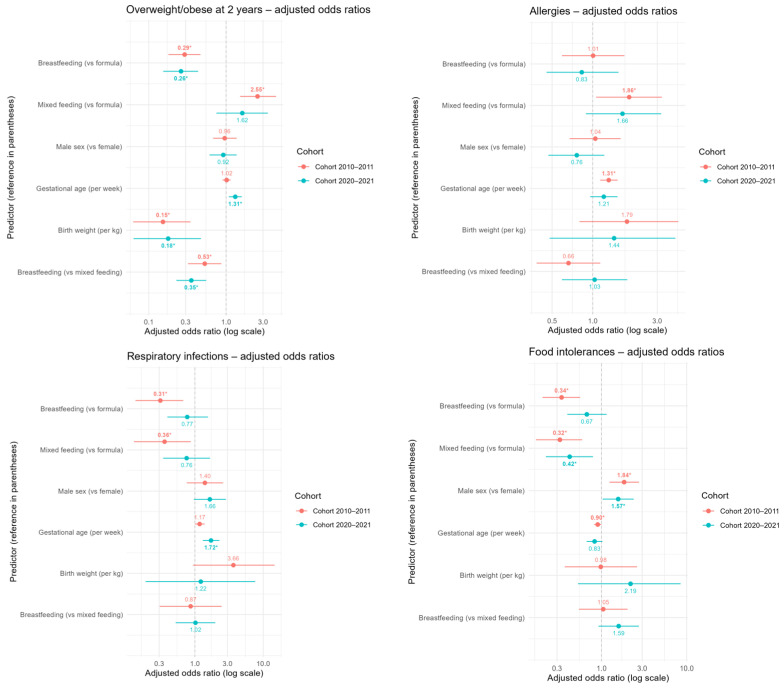
Logistic regression for predictors of outcomes: overweight/obesity (**top left**), allergies (**top right**), respiratory infections (**bottom left**), food intolerances (**bottom right**). Asterisks and bold OR values indicate predictors with *p* < 0.05.

The logistic regression shows that mixed feeding (vs. formula) was found to be a prediction factor for overweight/obesity at two years in the historic cohort [2010–2011—aOR 2.55 (95% CI: 1.52–4.43, *p* < 0.001); 2020–2021—aOR 1.62 (95% CI: 0.75–3.47, *p* = 0.215)], while breastfeeding acted as a protection factor against weight gain and the gender was not an influence (Figure 3). Both breastfeeding and mixed feeding appeared to be associated with lower odds of respiratory infections in the unadjusted analyses, particularly in the historical cohort. However, after covariate adjustment, breastfeeding was not associated with lower odds of respiratory infections in the 2010–2011 cohort [aOR 1.39 (95% CI 0.31–2.46)], indicating no protective effect. Mixed feeding (vs. formula) also slightly increased the odds for allergies in both groups [2010–2011—aOR 1.86 (95% CI: 1.06–3.25, *p* = 0.03); 2020–2021—aOR 1.66 (95% CI: 0.89–3.21, *p* = 0.119)]. Food intolerances had higher odds of occurring in males [2010–2011—aOR 1.84 (95% CI: 1.24–2.75, *p* = 0.003); 2020–2021—aOR 1.57 (95% CI: 1.03–2.39, *p* = 0.035)] and in breastfed infants (vs. mixed feeding) in the contemporary cohort (aOR 1.59, 95% CI: 0.92–2.74, *p* = 0.05).

## 4. Discussion

### 4.1. Fetal Growth Restriction and Risk of Obesity

Fetal growth restriction and low birth weight have been traditionally linked to increased perinatal morbidity. Accumulating evidence suggests they are also associated with accelerated postnatal growth and an increased risk of obesity, as well as obesity-related disorders (coronary disease, dyslipidemia, type 2 diabetes) during adulthood. This obesity trend, however, starts during early childhood, and most of the children and adolescents with obesity are more likely to also be obese as adults [17]. Excessive catch-up growth in infancy has been associated with later adverse cardiovascular and metabolic disorders [18,19]. In former FGR infants turned adolescents, postnatal catch-up growth is apparently responsible for increased BMI, visceral adiposity, and increased blood pressure [20].

The significantly higher birth weights observed in the contemporary cohort suggest either better intrauterine conditions or changes in obstetrical management. However, despite improvement, weight gain patterns remain altered, reiterating the idea that even milder degrees of fetal growth restriction may have long-term metabolic consequences. Also, metabolic programming in SGA infants is affected even when intrauterine conditions improve.

Our study confirms that former SGA infants remain a metabolically vulnerable population, even in the absence of neonatal complications. The rates of overweight and obesity at 2 and 3 years of age support the concept that postnatal growth acceleration begins early and may already reflect an alteration of metabolic programming. Although the prevalence of overweight slightly increased in the contemporary cohort, obesity rates decreased significantly, which may suggest a possible shift towards a more moderate catch-up growth pattern. This may stem from improvements in perinatal care, parental education, and feeding practices over ten years.

### 4.2. Infant Feeding

For a long time, breastfeeding has been recognized as the superior method of infant nutrition, with multiple health benefits for infants born with both normal and low birth weight [21].

One of the most relevant findings of our study is the protective role of breastfeeding against excessive weight gain, in contrast with the increased risk of obesity associated with mixed feeding.

Breastfeeding likely exerts this protective effect through several mechanisms: improved self-regulation of energy intake, hormonal influences, or modulation of the gut microbiome. In the systematic review by Resvick et al. [22], the authors mentioned the involvement of breastfeeding in catch-up growth, especially regarding length and cranial circumference, but to an extent that could not be determined.

Exclusive breastfeeding, for whatever duration, is consistently associated with lower risks of acute otitis media, asthma, and obesity [23], and it is considered to have a prophylactic effect on the development of allergies [24]. The increased percentage of exclusively breastfed SGA infants in our contemporary cohort is probably responsible for improved outcomes regarding allergies and obesity.

On an interesting note, mixed feeding was found to be a consistent predictor for overweight/obesity in both cohorts, even when compared to exclusive formula feeding. This somewhat counterintuitive and unexpected finding may stem from different possible causes, which are all plausible but remain speculative: inconsistent feeding patterns, overfeeding caused by overlapping feeding cues, and parental perception of breast milk insufficiency, leading to supplementation. Existing literature suggests several pathways that may contribute to this association. Bottle supplementation, which is common in mixed feeding, has been shown to reduce infants’ ability to self-regulate intake and is associated with higher milk consumption and more rapid weight gain [25]. Perceived insufficient milk supply—a frequent concern among breastfeeding mothers [26]—may prompt unnecessary supplementation, increasing total caloric intake. Furthermore, alternating between breast and bottle may introduce inconsistent feeding patterns [27], potentially disrupting appetite regulation and promoting non-responsive feeding behaviors. The association between mixed feeding and increased risk of overweight, which we observed in our study, warrants further investigation, as literature on mixed feeding is sparse and current evidence on this topic remains limited.

Our results highlight that mixed feeding is not metabolically neutral and should be more carefully addressed in clinical counseling.

### 4.3. Complementary Feeding

The World Health Organization recommends exclusive breastfeeding until 6 months of age, when complementary feeding should be initiated [28]. It is a recurrent recommendation for several years and the very strict time frame is well received by both family physicians and mothers in Romania. This was the main reason for its inclusion in our telephone interview. However, many medical societies believe this limit to be obsolete and does not account for the newest data we have on the development of allergies [29].

Appropriate complementary feeding was associated in our study with a lower incidence of allergies, supporting current evidence that both timing and diversity of food introduction play a role in immune tolerance development [30,31]. Recent studies have shown decreased rates of IgE-mediated allergies following early introduction of allergenic foods, regardless of specific feeding practices [32].

The lower incidence of allergies in the contemporary cohort may be a reflection of improved parental education and overall feeding practices, but the lack of statistical significance may appear for various reasons: other environmental or genetic factors may modulate this relationship, or parental reporting may introduce classification bias. Cultural beliefs are still very much ingrained for some, and social media exerts a formidable influence on others; therefore, in the absence of medical guidance regarding the proper introduction of allergenic foods, parents are reluctant to feed them to their infants prior to one year of age [33].

The marked increase in overall prevalence of food intolerances over one decade is noteworthy and may reflect either an increased awareness and diagnosis, or a true epidemiological rise determined by changes in dietary patterns.

The Western Diet Hypothesis states that modern diets consisting mainly of ultra-processed foods, refined sugars, saturated fats, and low levels of natural fiber can compromise intestinal health, even if breastfeeding and timing of complementary feeding are correct. A recent study from Brazil found that high maternal consumption of ultra-processed foods during lactation was associated with increased odds of infant malnutrition and stunting [34]. Increasingly younger children are exposed to ultra-processed foods as soon as weaning starts [35]. Also, the poor diversity of an infant’s microbiome, which aligns with the hygiene hypothesis, can impact intolerance rates that rise despite appropriate food introduction [36,37]. This lack of diversity disrupts the immune system’s ability to develop oral tolerance, causing it to overreact to harmless proteins in food even if they are introduced at the medically recommended times.

A study from 2024 linked postpartum maternal anxiety to lower dietary diversity in infants, as anxiety about potential reactions can lead to a more restricted variety of foods, under the guise of caution [38]. This paradoxically reduces the richness of the infant’s gut microbiome and increases intolerance risk. It may even be exacerbated in mothers of former SGA infants, often perceived as frail.

However, the apparent rise in food intolerances in our research must be interpreted with caution, as food intolerances were reported by parents, without verification against medical records or standardized diagnostic criteria. Therefore, several non-epidemiological explanations are equally plausible. Increased diagnostic awareness among healthcare providers, greater parental familiarity with the concept of food intolerance, and heightened healthcare-seeking behavior in the contemporary cohort may all contribute to higher reporting rates [39] in the absence of a true increase in prevalence. These factors may be particularly relevant given the growing public attention to food sensitivities and the widespread dissemination of related information through social media and parenting forums. Consequently, while dietary and microbiome-related mechanisms remain biologically plausible contributors, the observed trend may also reflect evolving perceptions and reporting practices rather than a genuine shift in disease burden.

One of the most surprising findings in our study was the very similar incidences of respiratory infections in children with both appropriate and inappropriate complementary feeding. This result is likely influenced by several factors: reporting bias (parents may underreport mild infections), recall bias (parents may not remember exactly the severity of infectious episodes), differences in healthcare-seeking behavior (parents with a stricter attitude towards health-related issues, such as appropriate complementary feeding may tend to overestimate the magnitude of respiratory symptoms) or potential confounding factors such as daycare attendance, siblings, or socioeconomic status, which we have not controlled in this study.

Given the strong body of evidence supporting the protective role of optimal nutrition against infections, this particular finding should be interpreted cautiously.

### 4.4. Timewise Comparison of Cohorts

The comparison between the historical and the contemporary cohort highlights encouraging trends, such as increased rates of breastfeeding, improved complementary feeding practices, and a reduced prevalence of obesity. These changes likely reflect both public health efforts and possibly updated pediatric and nutritional guidelines implemented over one decade. However, the persistence of overweight and the metabolic vulnerability of SGA infants indicate that current strategies remain insufficient, and more targeted interventions are needed.

### 4.5. Strengths and Limitations

One of the main strengths of our study is the large sample size, which allowed temporal comparisons through the dual-cohort design. Also, we were able to focus on a relatively homogeneous population (term singleton SGA infants without NICU admission).

An important topic that we were able to touch on is the issue of mixed feeding, which emerged in our study as a consistent risk factor across outcomes. This adds new data to the sparse body of evidence on this subject.

The main limitation we identified was the reliance on parent/caregiver-reported data, which may have introduced recall and reporting biases, as well as the lack of objective medical records for comorbidities. As a result, the classification of overweight/obesity relies on self-reported measurements, which are susceptible to social desirability bias. Prior research indicates that parents tend to inaccurately report weight and height in young children, potentially leading to under- or overestimation of BMI [40,41]. Because our dataset did not include information on the timing of the most recent clinical measurement, we were unable to perform a sensitivity analysis restricted to objectively measured values. Furthermore, Romania does not maintain a separate national pediatric growth reference, and WHO Child Growth Standards are used in clinical practice; therefore, benchmarking against national growth data was not feasible. These factors should be considered when interpreting the obesity-related findings. Also, the lack of data from children lost to follow-up in both cohorts could lead to selection bias. Another limitation could be the inclusion of late preterm infants (36-weekers), which may lead to confounding related to prematurity.

## 5. Conclusions

In conclusion, birth weight and early nutrition significantly shape growth patterns in former SGA infants. The prevalent role of mixed feedings in our considered outcomes, as well as the marked increase in food intolerances over one decade, is a surprising result that needs further attention. While improvements in feeding practices over time are encouraging, former SGA infants are still at increased risk for early-onset overweight and obesity. This underlines the need for targeted preventive strategies such as encouragement of exclusive breastfeeding, careful monitoring of mixed feeding, and parental education regarding complementary feeding.

## Figures and Tables

**Table 1:** Characteristics of the studied population.

	Group H 2010–2012	Group C 2020–2022	*p*
Total neonates	18,135	17,851	-
Total SGA (n; %)	912; 5.03%	937; 5.25%	0.446
Interviewed	600	508	-
GA (weeks)—mean (SD)	37.77 (2.80)	38.80 (1.01)	*p* < 0.001
BW (kg)—mean (SD)	2.31 (0.45)	2.73 (0.22)	*p* < 0.001
BL (cm)—mean (SD)	47.47 (3.08)	48.44 (1.74)	*p* < 0.001

Abbreviations: SGA—small for gestational age; GA—gestational age; BW—birth weight; BL—birth length; SD—standard deviation.

## Data Availability

The original contributions presented in this study are included in the article. Further inquiries can be directed to the corresponding author.

## Supplementary Materials

The following supporting information can be downloaded at: https://www.mdpi.com/article/10.3390/nu18142296/s1, Table S1: Variable-level missingness before imputation; Table S2: Gestational-age distribution by cohort; Table S3: Term-only sensitivity analysis: overweight/obese at 2 years; Table S4: Term-only sensitivity analysis: allergies; Table S5: Term-only sensitivity analysis: respiratory infections; Table S6: Term-only sensitivity analysis: food intolerances; Table S7: Observed allergy rates by complementary feeding type and cohort; Table S8: Adjusted association between inappropriate complementary feeding and allergies by cohort; Table S9: Formal interaction test.

## Abbreviations

The following abbreviations are used in this manuscript:

SGA	Small for Gestational Age
FGR	Fetal growth restriction
WHO	World Health Organization
CF	Complementary feeding
BMI	Body Mass Index
NICU	Neonatal Intensive Care Unit
