# Supplementary material for: Optimizing Feeding Practices Improves Outcomes During Early Childhood in Former Small for Gestational Age (SGA) Infants
Source: Nutrients. 2026 Jul 13;18(14):2296. doi: 10.3390/nu18142296 (PMC13415059; doi:10.3390/nu18142296)
Supplement: Supplementary file 1 [file nutrients-18-02296-s001.zip › nutrients-4385348-supplementary.pdf]

Supplementary files

Supplementary table S1. Variable-level missingness before imputation

Variable	Observed, n	Missing, n	Missing, %.
Weight status at 2 years	1081	27	2.4
Weight status at 3 years	1079	29	2.6
Food intolerances	968	140	12.6
Mode of delivery	966	142	12.8
Weight status at 4 years	905	203	18.3
Allergies	891	217	19.6
Respiratory infections	890	218	19.7
Feeding type	867	241	21.8
Complementary feeding	855	253	22.8
Environment	855	253	22.8

Supplementary table S2. Gestational-age distribution by cohort

Cohort	Total children, n	36 weeks, n (%)	≥37 weeks, n (%)
Cohort 2010–2011	600	148 (24.7%)	452 (75.3%)
Cohort 2020–2021	508	33 (6.5%)	475 (93.5%)

Supplementary table S3. Term-only sensitivity analysis: overweight/obese at 2 years

Predictor	aOR (95% CI) – 2010–2011	p – 2010–2011	aOR (95% CI) – 2020–2021	p – 2020–2021
Breastfeeding (vs formula)	0.33 (0.19–0.55)	p<0.001	0.26 (0.15–0.43)	p<0.001
Mixed feeding (vs formula)	2.01 (1.39–2.9)	p<0.001	1.15 (0.84–1.57)	p=0.380

Predictor	aOR (95% CI) – 2010–2011	p – 2010–2011	aOR (95% CI) – 2020–2021	p – 2020–2021
Male sex (vs female)	0.82 (0.53–1.27)	p=0.372	0.58 (0.31–1.09)	p=0.090
Gestational age (per week)	0.98 (0.81–1.20)	p=0.859	1.32 (1.09–1.62)	p=0.005
Birth weight (per kg)	0.05 (0.01–0.16)	p<0.001	0.10 (0.02–0.36)	p<0.001
Breastfeeding (vs mixed feeding)	0.49 (0.29–0.83)	p=0.008	0.35 (0.23–0.55)	p<0.001

Supplementary table S4. Term-only sensitivity analysis: allergies

Predictor	aOR (95% CI) – 2010–2011	p – 2010–2011	aOR (95% CI) – 2020–2021	p – 2020–2021
Breastfeeding (vs formula)	0.89 (0.52–1.55)	p=0.679	0.82 (0.45–1.54)	p=0.529
Mixed feeding (vs formula)	1.43 (1.18–1.74)	p<0.001	1.22 (0.98–1.52)	p=0.080
Male sex (vs female)	1.10 (0.69–1.72)	p=0.696	0.76 (0.47–1.23)	p=0.271
Gestational age (per week)	1.25 (1.02–1.52)	p=0.030	1.20 (0.95–1.52)	p=0.126
Birth weight (per kg)	2.26 (0.67–8.01)	p=0.194	0.58 (0.11–2.75)	p=0.513
Breastfeeding (vs mixed feeding)	0.62 (0.36–1.08)	p=0.094	0.99 (0.57–1.74)	p=0.981

Supplementary table S5. Term-only sensitivity analysis: respiratory infections

Predictor	aOR (95% CI) – 2010–2011	p – 2010–2011	aOR (95% CI) – 2020–2021	p – 2020–2021
Breastfeeding (vs formula)	0.21 (0.08–0.51)	p<0.001	0.72 (0.36–1.49)	p=0.368
Mixed feeding (vs formula)	0.33 (0.11–0.83)	p=0.027	0.74 (0.33–1.64)	p=0.453
Male sex (vs female)	1.39 (0.67–2.87)	p=0.370	1.72 (0.99–3.01)	p=0.054

Predictor	aOR (95% CI) – 2010–2011	p – 2010–2011	aOR (95% CI) – 2020–2021	p – 2020–2021
Gestational age (per week)	1.19 (0.88–1.60)	p=0.258	1.86 (1.40–2.51)	p<0.001
Birth weight (per kg)	5.17 (0.87–30.57)	p=0.070	1.18 (0.18–7.42)	p=0.859
Breastfeeding (vs mixed feeding)	0.65 (0.22–1.99)	p=0.454	0.98 (0.50–1.95)	p=0.963

Supplementary table S6. Term-only sensitivity analysis: food intolerances

Predictor	aOR (95% CI) – 2010–2011	p – 2010–2011	aOR (95% CI) – 2020–2021	p – 2020–2021
Breastfeeding (vs formula)	0.31 (0.18–0.54)	p<0.001	0.67 (0.40–1.15)	p=0.146
Mixed feeding (vs formula)	0.31 (0.15–0.60)	p<0.001	0.43 (0.23–0.82)	p=0.010
Male sex (vs female)	1.62 (1.01–2.62)	p=0.047	1.57 (1.03–2.40)	p=0.035
Gestational age (per week)	1.05 (0.85–1.29)	p=0.673	0.82 (0.66–1.01)	p=0.065
Birth weight (per kg)	1.90 (0.55–6.84)	p=0.319	2.20 (0.53–8.52)	p=0.262
Breastfeeding (vs mixed feeding)	1.01 (0.50–2.02)	p=0.988	1.55 (0.90–2.69)	p=0.115

Supplementary table S7. Observed allergy rates by complementary feeding type and cohort

Cohort	Allergies with appropriate CF, %	Allergies with inappropriate CF, %	p-value
Cohort 2010–2011	14.8	24.8	p=0.004
Cohort 2020–2021	21.8	29.0	p=0.118

Supplementary table S8. Adjusted association between inappropriate complementary feeding and allergies by cohort

Cohort	Predictor	aOR (95% CI)	p-value
Cohort 2010–2011	Inappropriate CF (vs appropriate CF)	2.99 (1.91–4.67)	p<0.001
Cohort 2020–2021	Inappropriate CF (vs appropriate CF)	2.83 (1.76–4.54)	p<0.001

Supplementary table S9. Formal interaction test

Test	N used in model	p-value
Cohort × complementary feeding interaction	1,108	p=0.868
